# Quantitative phosphoproteome analysis of embryonic stem cell differentiation toward blood

**DOI:** 10.18632/oncotarget.3454

**Published:** 2015-03-26

**Authors:** Manuela Piazzi, Andrew Williamson, Chia-Fang Lee, Stella Pearson, Georges Lacaud, Valerie Kouskoff, James A. McCubrey, Lucio Cocco, Anthony D. Whetton

**Affiliations:** ^1^ Cell Signaling Laboratory, Department of Biomedical Science (DIBINEM), University of Bologna, Italy; ^2^ Stem Cell and Leukaemia Proteomics Laboratory, Manchester Academic Health Science Centre, The University of Manchester, Manchester, UK; ^3^ Stem Cell Research Group, Manchester Academic Health Science Centre, University of Manchester, Manchester, UK; ^4^ Stem Cell Biology Group Paterson Institute for Cancer Research, Manchester Academic Health Science Centre, University of Manchester, Manchester, UK; ^5^ Department of Microbiology and Immunology, Brody School of Medicine at East Carolina University, Greenville, NC, USA

**Keywords:** hemangioblast, iTRAQ, phosphoproteomic, nucleus

## Abstract

Murine embryonic stem (ES) cells can differentiate *in vitro* into three germ layers (endodermic, mesodermic, ectodermic). Studies on the differentiation of these cells to specific early differentiation stages has been aided by an ES cell line carrying the Green Fluorescent Protein (GFP) targeted to the Brachyury (Bry) locus which marks mesoderm commitment. Furthermore, expression of the Vascular Endothelial Growth Factor receptor 2 (Flk1) along with Bry defines hemangioblast commitment. Isobaric-tag for relative and absolute quantification (iTRAQ^TM^) and phosphopeptide enrichment coupled to liquid chromatography separation and mass spectrometry allow the study of phosphorylation changes occurring at different stages of ES cell development using Bry and Flk1 expression respectively. We identified and relatively quantified 37 phosphoentities which are modulated during mesoderm-induced ES cells differentiation, comparing epiblast-like, early mesoderm and hemangioblast-enriched cells. Among the proteins differentially phosphorylated toward mesoderm differentiation were: the epigenetic regulator Dnmt3b, the protein kinase GSK3b, the chromatin remodeling factor Smarcc1, the transcription factor Utf1; as well as protein specifically related to stem cell differentiation, as Eomes, Hmga2, Ints1 and Rif1. As most key factors regulating early hematopoietic development have also been implicated in various types of leukemia, understanding the post-translational modifications driving their regulation during normal development could result in a better comprehension of their roles during abnormal hematopoiesis in leukemia.

## INTRODUCTION

Mouse embryonic stem cells (mESCs) are the *in vitro* counterparts of an *in vivo* population of cells specific to the early embryo within the inner cell mass. ES cells are pluripotent and different culture conditions can induce them to differentiate into the three primary germ layers (mesoderm, ectoderm and endoderm). In the mouse embryo around 6.5 days of gestation, epiblast cells migrate to form the primitive streak which contains the nascent mesoderm. Derived from mesoderm, hematopoiesis occurs in a consequent temporal pattern during embryonic development, around 7.5 days gestation in the blood islands in the yolk sac. Associated with this process is the formation of the hemangioblast population. This transient progenitor cell with the capacity to give rise to both endothelial and hematopoietic progenitors has been shown to be formed within the primitive streak [1].

mESCs have been studied extensively since they represent a potentially vast source of cells and tissues for regenerative medicine. Although there are data on the molecular processes governing the formation of hemangioblasts [2], specific phosphorylation events within the nucleus governing development have not yet been systematically analysed, and the mechanistic detail on how the hemangioblast is formed requires further analysis.

To further delineate the hemangioblast population, a reporter ES cell line with the GFP coding sequences targeted into the Brachyury (Bry) locus was created by Fehling et al [3]. ES cells can differentiate *in vitro* to form spheroid cultures called embryonic bodies (EBs). These structures contain the derivatives of all the three germ layers, and it is possible to track mesoderm and hematopoietic lineage commitment *in vitro* with the temporal expression of two specific genes, restricted to the lineage of interest. Bry, a member of the T-box gene family, is a marker for the early mesoderm formation (the majority of the cells in the primitive streak are Bry positive). Bry expression decreases when cells migrate away from the primitive streak and further differentiate. Flk1, the vascular endothelial growth factor receptor 2, identifies a mesodermal population of cells further committed for differentiation and is commonly expressed with Bry in populations with hematopoietic potential. The analysis of Bry and Flk1 expression allowed for the detection of three subpopulations: the Bry^−^Flk1^−^ population represents mES cells that have not yet undergone the differentiation process, thus resembling epiblast cells. The Bry^+^Flk1^−^ fraction corresponds to a population of early mesoderm differentiating cells. The Bry^+^Flk1^+^ fraction contains precursors for endothelial and hematopoietic lineages, the blast-colony forming cells (BL-CFC), representing the *in vitro* equivalent of the hemangioblast.

We have previously reported changes occurring in mES cells nuclear proteome during mesoderm-induced differentiation, using isobaric tags for relative and absolute quantification (iTRAQ^TM^) coupled to LC-MS/MS analysis [4]. This approach allowed a relatively deep proteomic penetration. However, activities of proteins are often regulated by translation and degradation rates, as well as by post-translational modifications, e.g. phosphorylation. It has been recently reported that post-translational modifications occur during ES cells differentiation, and they are also required for ES cell self-renewal [5]. Protein phosphorylation has been demonstrated to be involved in the regulation of many aspects of cellular functions including cell proliferation, differentiation, migration and signal transduction. It is likely that extracellular signals in part convey their signals to the nucleus to engender epigenetic changes to initiate altered gene transcription [6]. In the process of mesoderm formation some signaling molecules have defined roles in proliferation and development control, such as tumor growth factor beta, FGF, Wnt and Hedgehog families; in mouse, BMP4, Wnt3 or Nodal, are essential for mesoderm development [7–8]. Mice lacking canonical Wnt ligands do not develop the primitive streak and fail to generate mesoderm from the epiblast [9–11]. Therefore the Wnt signaling pathway is involved in the mechanism that induces mouse ES cells to differentiate, switching from self-renewal to differentiation. Phosphorylation mapping, by means of proteomic technologies, serves as a starting point for establishing a comprehensive database of the stem cell phosphoproteome. To define the phosphoproteome of hemangioblast cells, we compared it with that of epiblast-like and early mesoderm populations. Our observations provide potential new pathways for analysis of regulation in ES cell differentiation to the hemangioblast fate, that could also be unraveled in leukemias. Interestingly, recent findings propose a pivotal role for GSK3 and the chromatin remodeling factor Smarcc1/Baf155 in the decision fate of hematopoietic stem cells to differentiate. GSK3 is an important regulator of stem cell homeostasis, in particular ES cell self-renewal [12], but it also been recently implicated in leukemia stem cell physiology [13–14]. Smarcc1/Baf155 is necessary for heterochromatin formation and chromatin compaction during the differentiation process.

## RESULTS

### Phosphoproteomic analysis of early embryonic development

**Figure 1:**
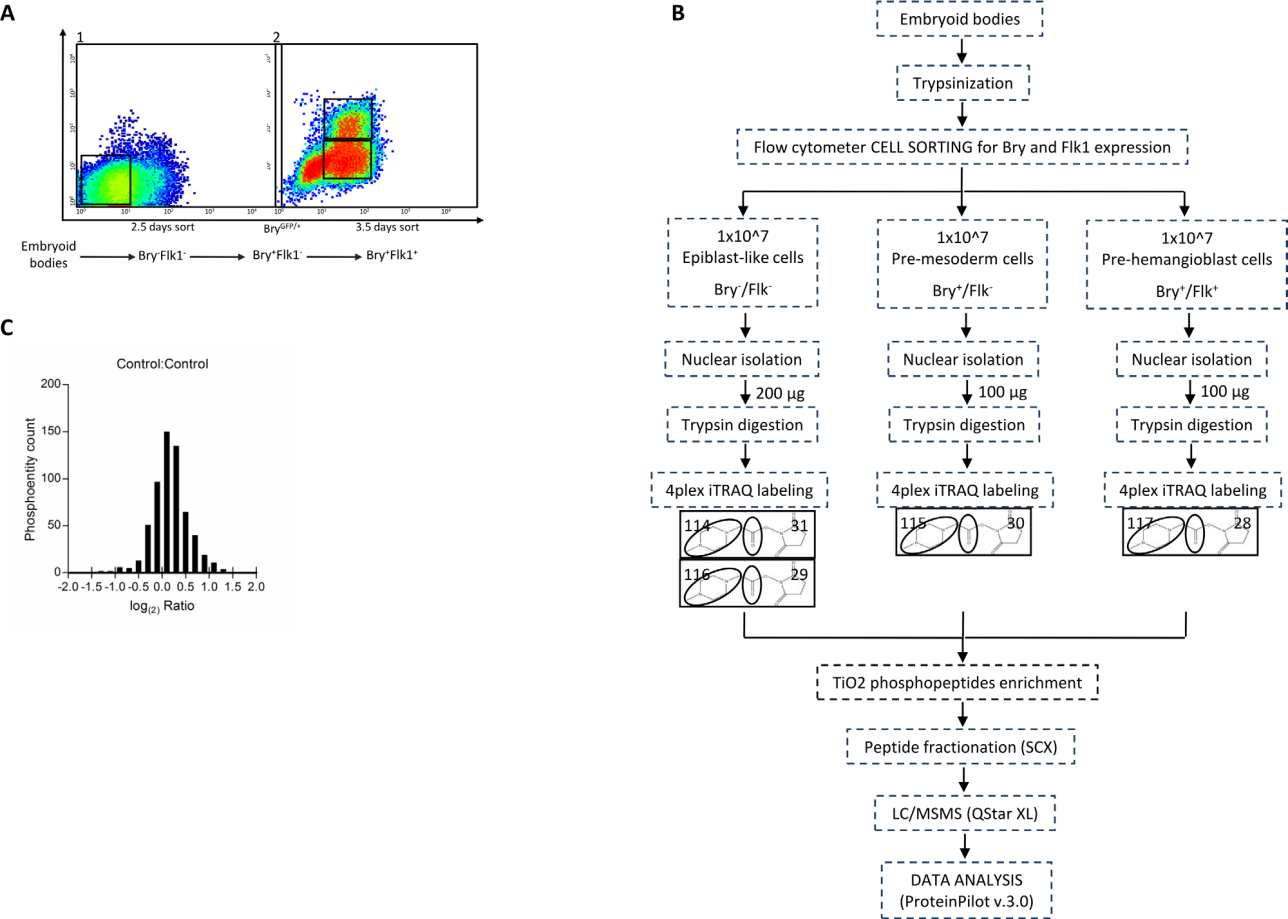
(A) Flow cytometric sorting of ES cells at specific time points of the hematopoietic differentiation for nuclear phosphoproteome analysis Differentiating ES cells were enriched based on Bry and Flk1 expression. 2 × 10^7^ cells were used for each experiment. At 2.5 days of differentiation, Bry^−^Flk1^−^ cells, corresponding to the epiblast-like stage, were collected by flow cytometric sorting (1). At 3.5 days two other populations, Bry^+^Flk1^−^ and Bry^+^Flk1^+^, corresponding to the mesoderm and hemangioblast (BL-CFC) cell populations, respectively, were collected (2). **(B)** Schematic workflow for the identification of changes in the phosphoproteome of mouse embryonic stem cells committed to the hematopoietic differentiation. Mouse embryonic stem cells sorted for Bry and Flk1 were generated from biological replicates. 100 μg of nuclear lysate were digested with trypsin to produce peptides that were labeled with 4-plex iTRAQ^TM^ reagent. Combined samples were then enriched for phosphopeptides via TiO_2_ column, separated with strong cation exchange chromatography (SCX) and subjected to nano-RPLC-MS/MS on a QTof mass spectrometer. **(C)** Distribution of the phosphoentity quantification ratios. Data normalization was obtained transforming all control ratios of phosphoentities identified with a sequence confidence above 20% with the logarithmic function at the base 2 (bins ranging from −2 to 2).

Once cultured without LIF and embryonic feeders, ES cells differentiate *in vitro* toward the mesoderm lineage. Three different populations, Bry^−^Flk1^−^, Bry^+^Flk1^−^ and Bry^+^Flk1^+^, were isolated from the differentiating ES cell culture as previously described [3] (Figure 1A). These distinct populations have been seen to represent epiblast, mesoderm committed and blast like colony forming (BL-CFC) cells. Approximately 1 × 10^7^ cells were collected for each population, nuclear enrichment was performed to concentrate the downstream proteomic analysis on pathways associated with regulation of gene expression (Figure S1). The same amount of each nuclear protein tryptic lysates (100 μg) was labeled with one of the 4-plex iTRAQ^TM^ channel reagents. All four reagents were used, allowing one internal control within the same LC-MS/MS experiment (Bry^−^Flk1^−^ nuclear lysate was labeled with two different iTRAQ^TM^ reagents) (Figure 1B). The samples were combined and enrichment for phosphopeptides performed using the TiO_2_ metal affinity enrichment approach. Samples were fractionated using SCX chromatography, before LC-MS/MS analysis. Data recorded from two independent biological replicates were analyzed using the Paragon Algorithm within the ProteinPilot^TM^ v.3.0. The total number of phosphopeptides identified was 2506, present in the two biological replicates, with 20% of confidence (Table S1). The data acquired showed a normal distribution between biological replicates with a modal value centered around 0 (1 in the linear scale) (Figure 1C).

### Relative quantification of phosphorylation changes occurring in the hemangioblast formation

Significance thresholds and quantitative phosphorylation changes were determined according to previous analysis [4–15–16]. To establish whether there was a change in peptide phosphorylation, only phosphopeptides exceeding 95% of the identifications compared to the control:control ratio (Bry^−^Flk1^−^: Bry^−^Flk1^−^) were considered. The interval of significance was calculated for each replicate based on the normal distribution of the internal control ratio (Figure 1C). Phosphorylation level was considered significantly changing when the fold change was < 0.6 (decrease) or > 1.5 (increase). Phosphopeptides with a ratio within the intervals of significance were manually removed from the data. Spectra were then manually checked to verify phosphorylation assignment, based on intact fragment presence, neutral loss of HPO_3_ (80 Da) or neutral loss of H_3_PO_4_ (98 Da). Using these stringent selection criteria 37 phosphoentities were of interest as they change during ES cell differentiation (see Table 1). With the exception of Vars, the phosphopeptides have been also identified in other phosphoproteomic analyses [17–20]. For only 9 phosphopeptides, the modification site and the protein kinase were determined using site-specific methods (referring to the phosphorylation site repository found on http://www.phosphosite.org/: 178226 non-curated sites, 103952 sites curated from literature) (Table 2). Regarding those peptides included in Table 1, epiblast cells shown 7 phosphoentities up-regulated and 14 phosphoentities down-regulated; early mesoderm cells exhibited 6 phosphoentities up-regulated and 12 phosphoentities down-regulated; in the hemangioblast 10 phosphoentities were up-regulated and 16 phosphoentities down-regulated.

**Table 1:** Phosphopeptides identified as changing in differentiating ES on analysis of iTRAQ^TM^ relative quantification

Acc.nb	Gene name	Protein name	Phosphopeptide sequence	Bry^+^Flk-: Bry-Flk-	Bry^+^Flk^+^: Bry^+^Flk^−^	Bry^+^Flk^+^: Bry^−^Flk^−^	GO Biological Process	GO Molecular Function
P57776	Eef1d	eEF1d protein	GATPAEDDE DKDIDLFG **pS**DEEEEDKEAAR	**1.5**	1.09	**1.61**	Positive regulation of NF-kB cascade	Signal transducer activity; Translational elongation factor activity
P57776	Eef1d	eEF1d protein	ATAPQTQ HV**pS**PMR	**1.96**	0.77	1.48	Positive regulation of NF-kB cascade	Signal transducer activity; Translational elongation factor activity
Q6J1H4	Utf1	UTF1	RLPAFSPP SPA**pS**PDAELR	**0.36**	**0.5**	**0.17**	Transcription, DNA-dependent	Transcription coactivator activity
Q6J1H4	Utf1	UTF1	RLPAF**pS**PPS PA**pS**PDAELR	**0.38**	**0.42**	**0.16**	Transcription, DNA-dependent	Transcription coactivator activity
Q6J1H4	Utf1	UTF1	RLPAFSPP **pS**PASPDAELR	**0.37**	**0.54**	**0.2**	Transcription, DNA-dependent	Transcription coactivator activity
Q6J1H4	Utf1	UTF1	RLPAFSPP **pS**PA**pS**PDAELR	**0.31**	**0.58**	**0.18**	Transcription, DNA-dependent	Transcription coactivator activity
Q6J1H4	Utf1	UTF1	SAGDVPVTTS DAFATSGGM AEPG**pS**PK	**0.39**	**0.48**	**0.19**	Transcription, DNA-dependent	Transcription coactivator activity
P17095	Hmga1	High mobility group protein HMG-I/HMG-Y	KQPPV **pS**PGTALVGSQK	1.22	**0.59**	0.73	Regulation of transcription; DNA-dependent, Negative regulation of cell proliferation	DNA-binding; Transcription coactivator activity
P52927	Hmga2	High mobility group protein HMG-A2	KQQQEPTCEP **pS**PKRPR	1.29	**1.89**	**2.38**	Positive regulation of stem cell proliferation	A-T DNA binding
Q9CQS8	Sec61b	Sec61 beta subunit	PGPTPSGTNVGS **pS**GRSPSK	1.07	**1.61**	**1.71**	Protein transport	Ribosome binding
Q9CQS8	Sec61b	Sec61 beta subunit	PGPTPSGTNV GSSGR**pS**PSK	1.1	**1.52**	**1.63**	Protein transport	Ribosome binding
P30999	Ctnnd1	Catenin delta-1 (p120 catenin)	GSLA**pS**LDSLRK	0.78	0.62	**0.48**	Wnt receptor signaling pathway; Regulation of transcription, DNA-dependent	Protein phosphatase binding; Protein kinase binding
Q8K019	Bclaf1	Bcl-2-associated transcription factor	IDI**pS**PSALRK	**0.53**	1.35	0.7	Regulation of transcription, DNA-dependent; Positive regulation of apoptosis	Protein binding; DNA binding
Q9WV60	Gsk3b	Glycogen synthase kinase 3b	GEPNV**pS**YICSR	**0.15**	1.48	**1.67**	Genetic imprinting; Multicellular organismal development; Wnt receptor signaling pathway	Protein kinase binding; Transferase activity
O88509	Dnmt3b	DNA (cytosine-5) methyltrans-ferase 3B	TTNDSAASE **pS**PPPKR	**0.38**	**0.51**	**0.19**	Methylation: Epigenetic	Transferase activity
Q01320	Top2a	Topoisomerase (DNA) II alpha	KPIKYLEE **pS**DDDDDLF	**0.52**	**1.58**	0.81	ATP catabolic process; Mitotic recombination; Embryonic cleavage	DNA topoisomerase activity; Chromatin binding
P14733	Lmnb1	Lamin B1	LKL**pS**PSPSSR	0.64	0.77	**0.48**	G2/M-specific positive regulation of cyclin-dependent protein kinase activity Positive regulation of JNK cascade	JUN kinase binding; Phospholipase binding
Q8BTI8	Srrm2	Serine/arginine repetitive matrix 2	MVQASSQSLLP PAQDRPR**p S**PVPSAFSDQSR	**0.59**	0.78	**0.46**	RNA splicing; mRNA processing	Protein N-terminus binding
Q569Z6	Thrap3	Thyroid hormone receptor associated protein 3	RIDI**pS**PSTFR	**0.56**	1	**0.55**	Steroid hormone receptor signaling pathway; Transcription - DNA dependent	Transcription coactivator activity; Nucleotide binding
P97496	Smarcc1	SWI/SNF complex 155 kDa subunit	RKP**pS**P **pS**PPPPTATESR	**1.6**	0.75	1.2	Chromatin remodeling; Organ morphogenesis; Transcription, DNA-dependent	Protein binding; DNA binding
Q8VDF2	Uhrf1	E3 ubiquitin-protein ligase UHRF1	RPLIA **pS**PSQPPPALR	0.82	0.7	**0.56**	Cell proliferation; Regulation of transcription, DNA-dependent; Multicellular organismal development	DNA binding; Methyl-CpG binding
P60904	Dnajc5	DnaJ (Hsp40) homolog, subfamily C, member 5	SL**pS**TSGESL YHVLGLDK	1.35	1.11	**1.51**	Protein folding; Negative regulation of neuron apoptosis	ATP-dependent protein binding; Heat shock protein binding; Unfolded protein binding
Q8K310	Matr3	Matrin-3	TE**pS**PAE GKEQEEK	**0.49**	1.16	**0.58**	Unannotated	Nucleic acid binding
Q9Z1Q9	Vars	Valyl-tRNA synthetase	L**pS**ATVTEAF VRLHEEGVI	1.44	**0.56**	0.79	tRNA aminoacylation for protein translation	Aminoacyl-tRNA ligase activity; Nucleotide binding
Q80XU3	Nucks1	Nuclear ubiquitous casein and cdk substrate	ATVTP **pS**PVKGK	**0.56**	0.85	**0.47**	Unannotated	DNA binding
Q6PR54	Rif1	Rap1-interacting factor 1	SSD**pS**VD IEEQEEK	0.69	**0.58**	**0.37**	Stem cell maintenance; cell cycle; Response to DNA damage stimulus	Protein binding
Q6PR54	Rif1	Rap1-interacting factor 1	VSDSSL **pS**PEK	0.84	0.64	**0.53**	Stem cell maintenance; cell cycle; Response to DNA damage stimulus	Protein binding
Q8CBW3	Abi1	Abl interactor 1	TNPPTQKPP SPPV**pS**GR	0.99	**1.67**	**1.64**	Peptidyl-tyrosine phosphorylation; Cell motility	Protein binding; Protein tyrosine kinase activator activity
Q08943	Ssrp1	FACT complex subunit SSRP1	EGINPGYD DYAD**pS**DE DQHDAYLER	**1.56**	0.68	1.05	Regulation of transcription, DNA-dependent	DNA binding
Q64012	Raly	hnRNP-associated with lethal yellow	GRL**pS**PVPVPR	**2.53**	**0.47**	**1.37**	RNA splicing; mRNA processing	RNA binding
Q9JIX8	Acin1	Acinus	HL**pS**HPEP EQQHVIQR	1.15	**0.52**	**0.59**	Apoptosis; Apoptotic chromosome condensation	Nucleic acid binding; Nucleotide binding
P11499	Hsp 90ab1	Heat-shock protein hsp84	IEDVG**pS**DE EDDSGKDK	0.55	1.35	0.73	Protein folding; Response to stress; Placenta development	Protein binding
P42208	Sept2	Septin 2 (NEDD5 protein)	IYHLPDAE **pS**DEDE DFKEQTR	1.01	**1.68**	**1.69**	Cell cycle	GTPase activity; Protein binding
O54839	Eomes	Eomes	KG**pS**PC AEEELPS AATAAATAR	**1.52**	0.76	1.15	Blastocyst development; Multicellular organismal development; Stem cell maintenance; Mesoderm formation; Endoderm formation; Interferon-gamma production	DNA binding; Transcription factor activity
Q9CW46	Raver1	Ribonucleoprotein PTB-binding 1	LL**pS**PIASNR	1.33	**0.49**	0.65	Unannotated	RNA binding
Q6P4S8	Ints1	Integrator complex subunit 1	LS**pS**TP PLSALGR	**2.45**	0.66	**1.62**	snRNA processing; Blastocyst growth; Inner cell mass cell proliferation; Apoptosis	Unannotated
Q8CGU3	Pnn	Pinin	RGF**pS**D SGGGPPAK	1.26	1.37	**1.71**	Cell-cell adhesion; Regulation of transcription, DNA-dependent; RNA splicing; mRNA processing	DNA binding

To be considered as changing, a phosphopeptide should have a ratio outside the range in which 95% of phosphopeptides ratios for the internal control replicates are found. Phosphopeptides shown are those where a confident assessment of a significant decrease or increase was found. To be included the data must have a -fold change <0.6 or > 1.5 with a *p* value of less than 0.05. The significant changes are shown in bold; the underlined values represent changes where the confidence levels were not met. Every spectrum has been manually checked to verify phosphorylation. The first three columns report the Accession Number (UniProtKB-SwissProt), the Gene and Protein name, and the primary sequence of the phosphopeptide identified, indicating in bold the aminoacid modified by phosphorylation and in brackets the phosphosite position in the full length protein. The next three columns report the log(2) of the iTRAQ ratios representing the changes in phosphorylation level occurring during the progression of the hematopoietic differentiation, comparing Bry^+^Flk1^−^:Bry^−^Flk1^−^, Bry^+^Flk1^+^:Bry^+^Flk1^−^ and Bry^+^Flk1^+^:Bry^−^Flk1^−^. The last two columns show the Biological process and Molecular function classification for the reported phosphopeptide-derived proteins, according to the Gene Ontology annotations. Data from two independent biological replicate were merged into the table.

**Table 2:** Phosphosite retrieved informations based on mass spectrometry identification and esperimental evidences

Acc.nb	Gene name	Protein name	Phosphopeptide sequence	(P)site position	MS evidence	EX evidence	Protein kinase
P57776	Eef1d	eEF1d protein	GATPAEDDED KDIDLFG**pS**D EEEEDKEAAR	S162	Y	Y	CK2
P57776	Eef1d	eEF1d protein	ATAPQTQ HV**pS**PMR	S133	Y	Y	Cdc2
Q6J1H4	Utf1	UTF1	RLPAFSPP SPA**pS**PDAELR	S18	Y	N	
Q6J1H4	Utf1	UTF1	RLPAF**pS**P PSPA**pS**PDAELR	S12, S18	Y	N	
Q6J1H4	Utf1	UTF1	RLPAFSPP **pS**PASPDAELR	S15	Y	N	
Q6J1H4	Utf1	UTF1	RLPAFSPP **pS**PA**pS**PDAELR	S15, S18	Y	N	
Q6J1H4	Utf1	UTF1	SAGDVPVT TSDAFATS GGMAEPG**pS**PK	S48	Y	N	
P17095	Hmga1	High mobility group protein HMG-I/HMG-Y	KQPPV **pS**PGTALVGSQK	S36	Y	Y	CDK2, HIPK2
P52927	Hmga2	High mobility group protein HMG-A2	KQQQEPT CEP**pS**PKRPR	S44	Y	Y	Cdc2
Q9CQS8	Sec61b	Sec61 beta subunit	PGPTPSGTN VGS**pS**GRSPSK	S14	Y	N	
Q9CQS8	Sec61b	Sec61 beta subunit	PGPTPSGTN VGSSGR**pS**PSK	S17	Y	N	
P30999	Ctnnd1	Catenin delta-1 (p120 catenin)	GSLA**pS**LDSLRK	S346	Y	N	
Q8K019	Bclaf1	Bcl-2-associated transcription factor	IDI**pS**PSALRK	S656	Y	N	
Q9WV60	Gsk3b	Glycogen synthase kinase 3b	GEPNV**pS**YICSR	S215	Y	N	
O88509	Dnmt3b	DNA (cytosine-5) methyltransferase 3B	TTNDSAASE **pS**PPPKR	S399	Y	N	
Q01320	Top2a	Topoisomerase (DNA) II alpha	KPIKYLEE **pS**DDDDDLF	S1521	Y	Y	PLK1, CK2
P14733	Lmnb1	Lamin B1	LKL**pS**PSPSSR	S392	Y	N	
Q8BTI8	Srrm2	Serine/arginine repetitive matrix 2	MVQASSQSLLP PAQDRPR**pS**P VPSAFSDQSR	S2404	Y	N	
Q569Z6	Thrap3	Thyroid hormone receptor associated protein 3	RIDI**pS**PSTFR	S679	Y	N	
P97496	Smarcc1	SWI/SNF complex 155 kDa subunit	RKP**pS**P**pS**P PPPTATESR	S327, S329	Y	N	
Q8VDF2	Uhrf1	E3 ubiquitin-protein ligase UHRF1	RPLIA**pS**PS QPPPALR	S289	Y	N	
P60904	Dnajc5	DnaJ (Hsp40) homolog, subfamily C, member 5	SL**pS**TSGES LYHVLGLDK	S10	Y	Y	AKT
Q8K310	Matr3	Matrin-3	TE**pS**PAEGKEQEEK	S619	Y	N	
Q9Z1Q9	Vars	Valyl-tRNA synthetase	L**pS**ATVTEA FVRLHEEGVI	N.R.	N	N	
Q80XU3	Nucks1	Nuclear ubiquitous casein and cdk substrate	ATVTP**pS**PVKGK	S181	Y	Y	CDK2
Q6PR54	Rif1	Rap1-interacting factor 1	SSD**pS**VDIEEQEEK	S1565	Y	N	
Q6PR54	Rif1	Rap1-interacting factor 1	VSDSSL**pS**PEK	S1683	Y	N	
Q8CBW3	Abi1	Abl interactor 1	TNPPTQKPP SPPV**pS**GR	S187	Y	N	
Q08943	Ssrp1	FACT complex subunit SSRP1	EGINPGYDD YAD**pS**DED QHDAYLER	S444	Y	N	
Q64012	Raly	hnRNP-associated with lethal yellow	GRL**pS**PVPVPR	S135	Y	Y	CDK2
Q9JIX8	Acin1	Acinus	HL**pS**HPEPEQQHVIQR	S710	Y	N	
P11499	Hsp90ab1	Heat-shock protein hsp84	IEDVG **pS**DEEDDSGKDK	S255	Y	Y	TK, CK2
P42208	Sept2	Septin 2 (NEDD5 protein)	IYHLPDAE **pS**DEDEDFKEQTR	S218	Y	Y	
O54839	Eomes	Eomes	KG**pS**PCAEE ELPSAATAAATAR	S117	Y	N	
Q9CW46	Raver1	Ribonucleoprotein PTB-binding 1	LL**pS**PIASNR	S576	Y	N	
Q6P4S8	Ints1	Integrator complex subunit 1	LS**pS**TPPLSALGR	S82	Y	N	
Q8CGU3	Pnn	Pinin	RGF**pS**DSGGGPPAK	S66	Y	N	

The first three columns report the Accession Number (UniProtKB-SwissProt), the Gene and Protein name, and the primary sequence of the phosphopeptide identified, indicating in bold the aminoacid modified by phosphorylation and in brackets the phosphosite position in the full length protein. The next three columns report the phosphosite position with respect to the protein primary sequence, whether the site has been identified by mass-spectrometry based methods (MS evidence) and experimental methods (EX evidence). Y = yes, N = no. These informations were retrieved from PhosphositePlus; experimental methods include aminoacid sequencing, site-directed mutagenesis, modification site-specific antibodies and targeted MS strategies. The last column report the protein kinase responsible for the specific site phosphorylation, identified by EX evidences.

We focused the data analysis on early phosphorylative changes, occurring in the first stages of the differentiation process, proceeding from the epiblast to the early mesoderm cells (Bry^+^Flk1^−^ and Bry^−^Flk1^−^), and those which are differentially regulated in the BL-CFC cells (Bry^+^Flk1^+^). According to this analysis, phosphopeptides can be divided into 3 groups. Group 1: phosphopeptides up- or down-regulated during early mesoderm differentiation. Group 2: phosphopeptides up- or down-regulated in BL-CFC cells. Group 3: phosphopeptides changing neither in Bry^−^Flk1^−^, Bry^+^Flk1^−^ nor Bry^+^Flk1^+^. Interestingly, almost half of the phosphopeptides that were differentially regulated in the Bry^+^Flk1^−^ and Bry^−^Flk1^−^, were not changing in the Bry^+^Flk1^+^ and *vice versa*. Only phosphopeptides emanated from Ef1d and Ints1 were up-regulated either in the progression from Bry^−^Flk1^−^ to Bry^+^Flk1^−^ and in the Bry^+^Flk1^+^; Utf1, Dnmt3b, Srrm2 Thrap3, Matr3 and Nucks1 phosphopeptides were instead down-regulated in both stages. Only phosphopeptides belonging to the proteins Hmga1, Vars and Raver1 were neither changing in the progression Bry^−^Flk1^−^ to Bry^+^Flk1^−^ and in the hemangioblast cells, indicating that post-translational regulation involving these proteins may be fundamental for the transition from the Bry^+^Flk1^−^ to Bry^+^Flk1^+^.

### The regulation of consequential temporal processes, by means of phosphorylation, governed the hemangioblast development

**Figure 2:**
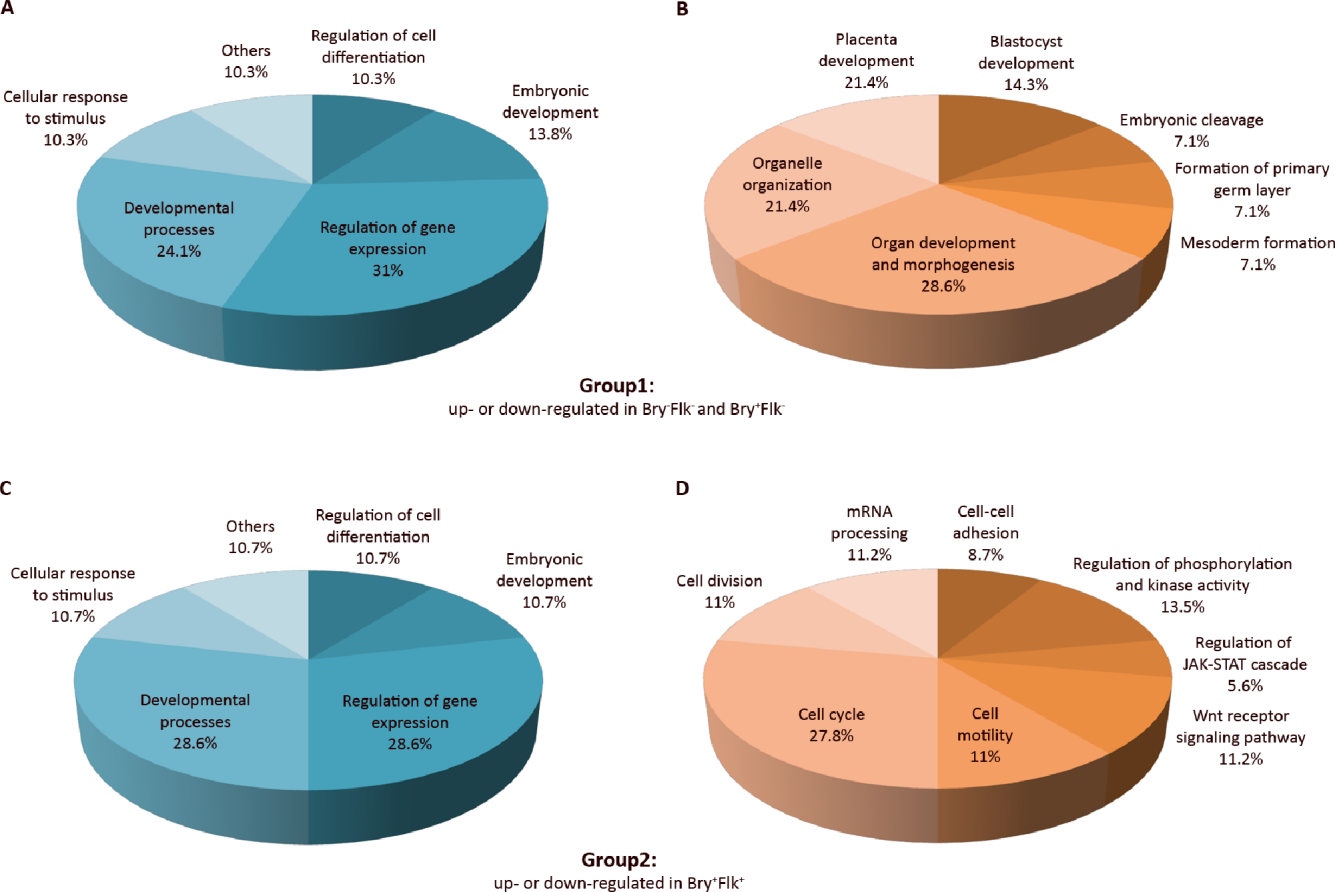
Biological classification of differentially regulated phosphopeptides Pie chart representation of the phosphopeptide-emanated proteins classified into groups by Biological process. Phosphopeptides from Group 1 (up- or down-regulated in Bry^−^Flk1^−^ and Bry^+^Flk1^−^) and Group 2 (up- or down-regulated in Bry^+^Flk1^+^) were considered as they are indicative for the regulation of stem cell differentiation. The relative frequency of over-represented categories is reported as percentage in the pie charts. **(A)** and **(C)** Biological processes of phosphopeptides differentially regulated both in Group 1 and Group 2, respectively. **(B)** and **(D)** Biological processes specific to phosphopeptides differentially regulated either in Group 1 or Group 2. Gene Ontology classification was performed using the plugin BiNGO v2.42 within Cytoscape v.2.8.2, assessing over-represented categories with a hypergeometric statistical test and Benjamini & Hochberg False Discovery Rate correction (*p* < 0.05). As a reference set, the whole mouse repository annotation was used.

Comparing the phosphopeptides changing in Bry^+^Flk1^+^ and those which occur in the very early steps of differentiation, according to the Gene Ontology, different categories of biological processes were involved (Figure 2). Proteins involved in regulation of cell differentiation and gene expression, developmental processes and especially embryonic development, such as Ints1, Top2a, Dnmt3b and Gsk3b, Utf1, Bclaf1, Thrap3 and Ssrp1, were found to be differentially phosphorylated in all the three stages of ES cells differentiation (Figure 2A and 2C). Phosphorylation on Ints1 and Dnmt3b was regulated the same in Bry^+^Flk1^−^:Bry^−^Flk1^−^ and Bry^+^Flk1^+^:Bry^−^Flk1^−^; Top2a decreased in Bry^+^Flk1^−^:Bry^−^Flk1^−^, but it didn't change in the Bry^+^Flk1^+^ while Gsk3b phosphorylation decreased as cells started to differentiate, then progressively increased as cells became Bry^+^Flk1^+^.

However, as expected, there are processing taking place in a consequential temporal pattern. As mES cells started to differentiate, phosphopeptides significantly changing were emanated from proteins involved in blastocyst and placenta development, the formation of the primary germ layer and mesoderm; such as Eomes, Hsp90ab1, Smarcc1 and Gsk3b. Conversely, as the BL-CFC cells were formed, differentially phosphorylated peptides emanated from proteins involved in cell cycle, cell division and mRNA processing such as Uhrf1, Sept2, Rif1, Hmga2, Srrm2 and Pnn; in the regulation of phosphorylation and kinase activity, such as the Wnt receptor signaling pathway (Ctnnd1 and Abi1) and the JAK-STAT cascade (Hmga2). Notably, few proteins also had a role in cell-cell adhesion and cell motility, such as Ctnnd1, Pnn and Rif1.

### Comparison with expression dataset

**Figure 3:**
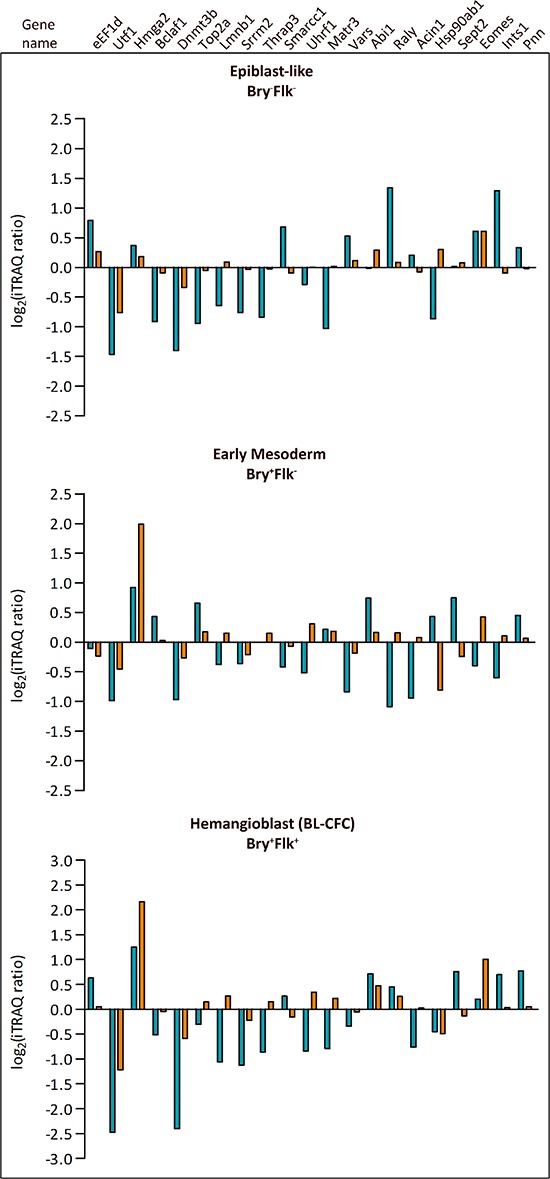
Comparison between phosphopeptides and protein expression levels Panels show changes in identified phosphopeptides compared to the expression profile of the referring proteins previously identified [4]. *Blue histograms* represent phosphorylation and *orange histograms* refer to protein expression level changes in Bry^−^Flk1^−^ (Epiblast-like cells), Bry^+^Flk1^−^ (Early mesoderm) and Bry^+^Flk1^+^ (Hemangioblast).

Twenty-one phosphopeptides within this dataset were found to emanate from proteins analyzed in the nuclear proteome expression analysis associated with Bry and Flk1 expression previously published [4] (Figure 3). Although the expression of the proteins did not change during hematopoietic differentiation, 9 phosphopeptides displayed differential regulation: Hmga1, Sec61b, Ctnnd1, Gsk3b, Dnajc5, Nucks1, Rif1, Ssrp1, Raver1. Among these, phosphorylation on Hmga1 and Raver1 only decreased in the progression from Bry^+^Flk1^−^ to Bry^+^Flk1^+^; while Raver1 is involved in the regulation of alternative splicing [21], Hmga1 plays a role in modulation of gene expression during development and embryogenesis, and its expression is known to be markedly diminished in differentiated cells [22]. Interestingly, Ctnnd1, Gsk3 and Rif1, are involved either in the regulation of cell differentiation and developmental processes, but also in the regulation of the cellular response to signaling pathways, such as the Wnt receptor signaling pathway. Moreover, GSK3 was found to play a pivotal role in controlling the decision fate of ES cells between self-renewal and differentiation; in fact inhibition of GSK3 has been found to promote and maintain mESC self-renewal [23]. As shown in Figure 3, in the majority of the cases normalization against protein level revealed no protein level change modulating stoichiometry of phosphorylation. For example, Hmga2 expression was augmented as ES cells differentiate and it remains high in the Bry^+^Flk1^+^: we found a peptide whose phosphorylation increased as mES cells differentiate, and this phosphorylation is maintained in the Bry^+^Flk1^+^ population. Hmga2 can exert a negative regulation on the Jak-Stat signaling cascade [24]. The Jak/Stat pathway promotes ESCs self-renewal [25] and is required for self-renewal of *Drosophila* sperm stem cells [26]. Conversely, only few phosphopeptides did not exhibit a correspondence between phosphorylation and protein expression. Among these, is the phosphopeptide emanating from the chromatin remodeling factor Smarcc1/Baf155, whose phosphorylation increased as mESCs differentiated toward mesoderm lineage commitment. Interestingly, Schaniel et co-workers [27], showed that Smarcc1 plays a balance between gene repression, which maintains ESCs in the self-renewal state and chromatin rearrangements, that led to the expression of genes involved in differentiation.

## DISCUSSION

Phosphoproteomic data can be a powerful tool to broaden understand of ES cells and how their ultimate fate is determined. Many transcription regulators such as epigenetic and transcription factors, as well as a large number of kinases are phosphorylated in embryonic stem cells [18–28, 29], suggesting that these post-translational modifications play a key role in determining stem cell fate. Our study reported the identification of novel phosphosites within proteins involved in ES cell mesoderm differentiation and the relative quantification of the phosphorylation stoichiometry during the progression from epiblast-like cells to the hemangioblast (BL-CFC cells).

The Gene Ontology classification highlighted that different biological processes play a role during the mesoderm-induced mESCs differentiation. During differentiation, phosphopeptides emanated from proteins involved in embryonic development were identified. In the last stage, when the hemangioblast is formed, phosphorylation differentially influences proteins involved in cell cycle, regulation of transcription and signaling pathways, indicating that mass spectrometry and phosphoproteomic analysis identified key modulators of the development process toward blood. For example, we reported a phosphopeptide emanated from Uhrf1 (also called Np95), which is known to contain a cyclin A/E-Cdk2 phosphorylation site. It has been demonstrated not only that Uhrf1 is essential for maintaining genomic methylation in embryonic stem cells by recruiting DNA methyltransferase (Dnmt) 1 to hemi-methylated CpG sites generated during replication [30], but also to interact with *de novo* methyltransferases Dnmt3a and Dnmt3b, mediating promoter silencing before DNA methylation is detected [31]. Uhtf1 is also involved in the regulation of Top2a. It has been suggested that Uhrf1 regulates transcription of Top2a by binding to an inverted 5′-CCAAT-3′ box in the Top2a promoter and activating expression [32]. Moreover it has been established that Uhfr1 contributes to genomic stability functioning in the response pathways against DNA damage and replication arrest [33]. Phosphopeptides emanated from both Top2a and Dnmt3b were found within our dataset.

Of particular interest was the identification of a phosphopeptide emanated from GSK3b, which phosphorylation increases as stem cells differentiate. GSK3b activity has been demonstrated to be crucial for the switch between stem cell self-renewal and differentiation. The regulation of GSK3 activity is able to inhibit self-renewal and promote differentiation [34]. But GSK3 functions in a wide range of cellular processes and it can both act as a tumor suppressor and as oncogene, in the development of various human cancers [35–37]. Of the phosphopetide emanated proteins within our dataset, 7 proteins present with at least 1 potential site that can be phosphorylated by GSK3b with high stringency (http://scansite3.mit.edu) (Acin1, Blacf1, Dnmt3b, Pnn, Rif1, Srrm2, Thrap3). Only 1 of the phosphopeptides identified contain the putative GSK3b consensus sequence (Abi1, medium stringency).

Another interesting protein was the Smarcc1/Baf155, that we found to be phosphorylated on S327 and S329; the phosphorylation of this doubly phosphorylated peptide decreased as stem cells differentiate toward the mesoderm commitment. Smarcc1/Baf155 belongs to the SWI/SNF multiprotein chromatin remodeling complex, and is required for heterochromatin formation and chromatin condensation during differentiation. In human ES cells, Smarcc1/Baf155 plays a dual role in maintaining pluripotency: it can promote or repress self-renewal gene expression, e.g. Nanog [38]. Furthermore, the SWI/SNF chromatin-remodeling complex also promotes somatic cell reprogramming by facilitating Oct4 binding to its target promoters. In retinoic acid-mediated differentiation, Baf155 can integrate p53 function to regulate ectodermal and mesodermal gene expression [27]. The possibility of Aurka-mediated p53 phosphorylation altering the interaction between p53 and the SWI/SNF complex as well as other epigenetic regulatory complexes is particularly tantalizing and may, in part, explain the apparent paradoxical requirement for SWI/SNF in both ESC maintenance and differentiation. As *in silico* analysis suggests (http://scansite3.mit.edu), Erk1 might be the kinase deputated to phosphorylate S327 and S329. Recently, Erk1/2, as GSK3b, was found to promote self-renewal, maintaining cellular growth capacity and reinforcing suppression of commitment [39–40].

We are only beginning to understand the molecular and cellular mechanisms of normal hematopoietic development and many pieces of these puzzle are still missing. The *in vitro* differentiation of ES cells has been instrumental in the identification and characterization of the hemangioblast.

Understanding how blood cells are generated is important from a biological perspective but also has potential implications in the treatment of blood diseases. Searches for post-translational regulatory processes are likely to lead to the identification of new important players in blood specification than can be altered in blood cancers. Knowing how signaling molecules regulates, by phosphorylation, their downstream targets could represent a precise stem cell signaling process that can lead to blood cancers, if unregulated or disrupted.

## MATERIAL AND METHOD

### Cell culture and differentiation

The Bry^+^ ES cell line was generated by Fehling et al. [3], introducing the GFP gene into one allele of the Bry (Brachyury) gene of the mouse embryonic stem cell line clone E14.1. ES cells were maintained and differentiated as previously described [3].

### Cell sorting flow cytometry

EBs were harvested, trypsinized and the single cell suspension analyzed on a FacsCalibur flow cytometer (BD Bioscences). Staining and isolation of cell populations were performed as previously described [3]. The expression of Bry with the Flk1 receptor in a temporal pattern revealed the emergence of three distinct populations, Bry^−^Flk1^−^, Bry^+^Flk1^−^, Bry^+^Flk1^+^, which represented a developmental progression from epiblast-like to early mesoderm then BL-CFC cells [3]. These sorted populations were used for analysis of the phosphoproteome.

### Nuclear isolation

Nuclear extraction was performed using the Nuclear Extract kit (Active Motif, Brussels, Belgium) as previously described [41]. Briefly, 1×10^7^ cells from each sorted population were washed in ice cold Hanks buffer (136.7 mM NaCl, 5.35 mM KCl, 0.812 mM MgSO_4_, 1.28 mM CaCl_2_, 5.5 mM Glucose, 24 mM Hepes, pH 7.4) with phosphatase inhibitors and then lysed in hypotonic buffer, following the manufacturer's instructions. Nuclei were then lysed in 1 M triethylammonium bicarbonate lysis buffer (TEAB, Sigma-Aldrich, St. Louis, MO), 0.05% (w/v) SDS, protease inhibitor cocktail, phosphatase inhibitors and Benzonase (Novagen, UK). The protein concentration was determined using the Bradford protein assay (Bio-Rad, Hemel Hempstead, UK). The nuclear purity was assessed by Western Blot analysis for nuclear (lamin a/c) and cytoplasmic (β-tubulin) markers.

### 4-plex iTRAQ^TM^ labeling

An aliquot of 100 μg of each nuclear protein sample was reduced, digested and labeled with four-channel iTRAQ^TM^ reagent (Applied Biosystem, Framingham, MA) according with the manufacturer's instructions. As previously described [41], protein were reduced with 50 mM tris-(2-carboxyethyl)phosphine, then alkylated by adding 200 mM methylmethanethiosulphate. Trypsin (5 μg, 0.5 μg/μl, Promega) was added, and the samples were incubated overnight at 37°C. After cooling to room temperature, peptides were dried, reconstituted in 20 μl TEAB and labeled with iTRAQ^TM^ reagent. In all experiments isobaric labeling exceeded 99% of total identified peptide using mass spectrometry. The four isobarically labeled reactions were pooled together and dried down.

### TiO_2_ phospho-enrichment

The iTRAQ^TM^ labeled peptide mixture was resuspended in 150 μl of lactate loading buffer (240 mg/ml lactate in 80% (v/v) Acetonitrile, 1% (v/v) Trifluoro-acetic acid (TFA). TiO_2_ columns (TopTip, Glygen, Corp, USA) were equilibrated with 150 μl of lactate loading buffer then the samples loaded. The tip was washed twice with 60 μl of lactate loading buffer and four times with 60 μl of wash buffer (80% (v/v) Acetonitrile, 5% (v/v) TFA). Bound peptides were then recovered by elution in 60 μl of elution buffer (ammonium water [20 μl NH_3_ in 980 μl H_2_O], pH 10.5). samples were then concentrated to a few μl in a SpeedVac prior to Strong Cation Exchange (SCX) chromatography.

### SCX fractionation

Enriched phosphopeptides were fractionated off-line using an SCX cation exchange column (10 cm × 2.1 mm PolyLCPolysulfoethyl A column, 5 μm beads, 200 μÅ pore size (The Nest Group, Southborough, MA, USA) on a LC Packings Ultimate LC system running at 250 μl/min. Peptides were diluted 10-fold in SCX loading buffer (10 mM KH_2_PO_4_ in 25% (v/v) ACN, pH 2.7). The eluate was collected in 1 min intervals over the following gradient of increasing salt concentration: a 45 min linear gradient of 0–250 mM KCl, followed by a 10 min linear gradient of 250–500 mM KCl, then 5 min of 1 M KCl. Each fraction was then lyophilized in a SpeedVac concentrator (Labconco).

### Mass spectrometry

Peptides were analyzed using a QStar^®^ XL mass spectrometer (AB Sciex, Warrington, UK) as previously described [41]. Briefly, dried peptide fractions were resuspended in 120 μl of Buffer A (2% (v/v) acetonitrile 0.1% (v/v) formic acid). For each analysis 60 μl of sample was loaded onto a on-line column (15 length;75 μm inner diameter) packed with RP C_18_ PepMap100 (3 μm, 100 A) using a Ultimate pump (LC Packings, Amsterdam, Netherlands) and separated over a 120 min solvent gradient from 5.9% (v/v) acetonitrile/0.1% (v/v) formic acid to 41% (v/v) acetonitrile/0.1% (v/v) formic acid coupled to a QStar^®^ XL mass spectrometer (AB Sciex, Warrington, UK). Data were acquired using an information dependent acquisition (IDA) designed with Analyst QS 2.0 (AB Sciex, Warrington, UK) where, for each cycle, the two most abundant multiply charged peptides (2+ to 4+) above a 20 count threshold in the MS scan with m/z between 400 and 2000 were selected for MS/MS. Each ion was selected a maximum of two times, and then dynamically excluded (± 50 mmu) for 40 seconds.

### Data analysis

Spectra were processed using ProteinPilot^TM^ v.3 software (Paragon algorithm v.3.0.0.0, AB Sciex, Warrington, UK), searching against a mouse Celera Discovery System database (mouse_KBMS5_0_20050302, 115.660 entries). Default search parameters were set with MS tolerance of 0.5 Da, MS/MS tolerance of 0.1 Da. Identification focus was on biological modifications with phosphorylation emphasis as a special factor. Search parameters include methylmethanethiosulfonate (MMTS) alkylation of cysteine, iTRAQ^TM^ modification of lysine and N-terminal residues. The searched parameter was set for reverse searching for false discovery, so that at the 95% confidence level there is a false positive identification rate of < 1%. Ratio values for each phosphoentity were obtained from weighted averages of multiple spectra when appropriate, similarly to the way ProteinPilot^TM^ calculates ratios for proteins. Phosphopeptides identified with a confidence ≥ 20 were included in the analysis. ProteinPilot^TM^ biases were used to correct for any sampling error so that the median value of log2(ratios) of the phosphoentity distribution is equal to 0 for each pair of iTRAQ^TM^ reagents. The distribution of the biological replicates Control(Bry^−^Flk^−^):Control(Bry^−^Flk^−^) was used to determine the biological variability of the procedure by combining the 2 control ratios. Student's *t* test was performed between the values of 2 biological replicates for each appropriate ratio and the Gaussian distribution of the biological replicates. Phosphoentities with a Student's *t*-test *p* value < 0.05 were considered as potentially changing. Phosphoentities changing between the controls, i.e. for Bry^−^Flk^−^:Bry^−^Flk^−^ ratio, with a *p* value < 0.05 were manually removed from the analysis. All the phosphorylation events were validated manually using the spectral data acquired. The phospho-dataset was then compared with the dataset obtained from the total nuclear expression analysis [4].

## SUPPLEMENTARY FIGURES AND TABLES




