# Supplementary material for: Quantitative phosphoproteome analysis of embryonic stem cell differentiation toward blood
Source: Oncotarget. 2015 Mar 26;6(13):10924–39. doi: 10.18632/oncotarget.3454 (PMC4484429; doi:10.18632/oncotarget.3454)
Supplement: Supplementary file 1 [file oncotarget-06-10924-s001.pdf]

SUPPLEMENTARY FIGURE

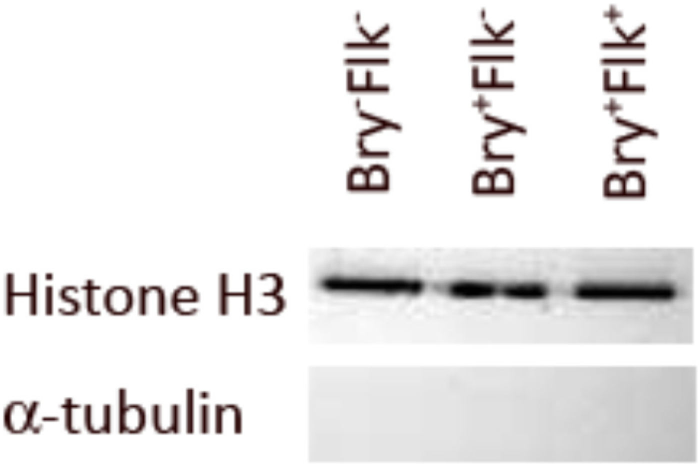

Supplementary Figure S1: Western blot analysis of known nuclear and cytosolic markers in fractions prepared from ES cells. ES cells were differentiated, sorted and a nuclear fraction was prepared, as described in Material and Methods. Nuclear lysates (20 µg) from Bry^-Flk^-, Bry^+Flk^- and Bry^-Flk^+ cells, were separated in a 4%–20% gradient SDS-PAGE, and immunoblotted with specific antibodies directed against a cytosolic (α-tubulin) and a nuclear (histone H3) protein, to assess the purity of nuclear preparation enrichment.
